# Relating Habitat and Climatic Niches in Birds

**DOI:** 10.1371/journal.pone.0032819

**Published:** 2012-03-12

**Authors:** Jean-Yves Barnagaud, Vincent Devictor, Frédéric Jiguet, Morgane Barbet-Massin, Isabelle Le Viol, Frédéric Archaux

**Affiliations:** 1 Irstea, Domaine des Barres, Nogent-sur-Vernisson, France; 2 Institut des Sciences de l'Evolution, UMR CNRS-UM2 5554, Montpellier, France; 3 Muséum National d'Histoire Naturelle, UMR 7204 MNHN-CNRS-UPMC, CP 51, Paris, France; University of Lausanne, Switzerland

## Abstract

Predicting species' responses to the combined effects of habitat and climate changes has become a major challenge in ecology and conservation biology. However, the effects of climatic and habitat gradients on species distributions have generally been considered separately. Here, we explore the relationships between the habitat and thermal dimensions of the ecological niche in European common birds. Using data from the French Breeding Bird Survey, a large-scale bird monitoring program, we correlated the habitat and thermal positions and breadths of 74 bird species, controlling for life history traits and phylogeny. We found that cold climate species tend to have niche positions in closed habitats, as expected by the conjunction of the biogeographic history of birds' habitats, and their current continent-scale gradients. We also report a positive correlation between thermal and habitat niche breadths, a pattern consistent with macroecological predictions concerning the processes shaping species' distributions. Our results suggest that the relationships between the climatic and habitat components of the niche have to be taken into account to understand and predict changes in species' distributions.

## Introduction

Biogeography and community ecology are being increasingly integrated into a common framework in which the interaction between local and large-scale processes are recognized as influencing community dynamics [1]. Current global changes affect species' distributions at various scales, from the turnover of local communities due to habitat modifications [2] to shifts in species ranges in response to climate change [3]. In particular, rapid changes in climate conditions and habitat suitability have been largely recognized as two major threats to biodiversity [4]. Yet, predictions of distributional responses to such multifaceted and multi-scale changes are hindered by the multidimensionality of the ecological niche. How the various dimensions of the niche together shape species' distribution patterns at various spatial scales is thus of fundamental interest to ecology and conservation biology [5].

**Figure 1:**
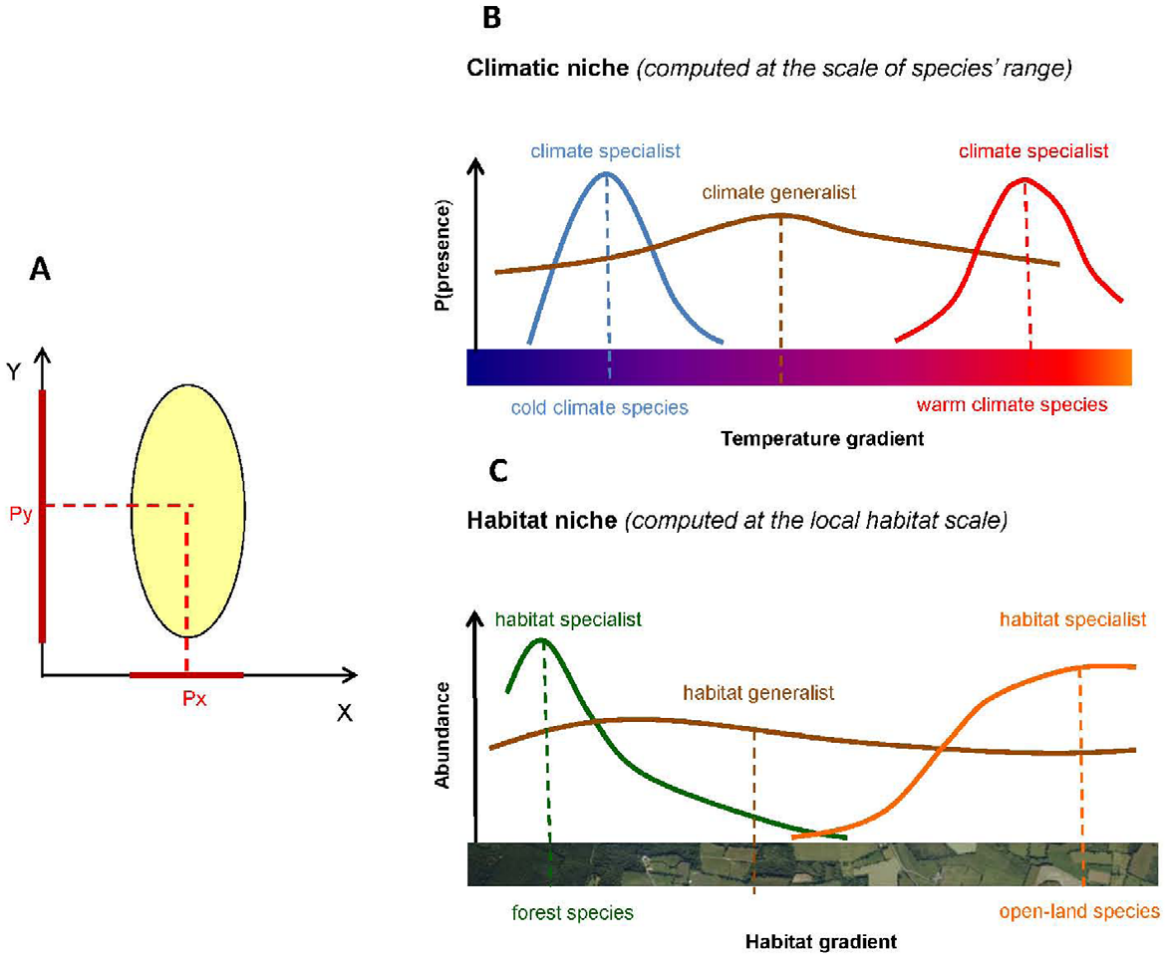
Definition of the ecological niche used in this article. (**A**) The environmental space can be represented as a set of axes (here, two: *X*, *Y*), each representing a gradient of resource or condition. A species' niche is defined as the range of each of these gradients that the species can exploit/occupy/cope with (yellow ellipse). The projection of the niche on each gradient is defined by a position (*P_x_*, *P_y_*) and a breadth (red solid lines). In our analyses, we consider two axes: (**B**) **a thermal axis** (referred to in the text as ‘thermal niche’) corresponds to a gradient of temperature; (**C**) **a habitat axis** (‘habitat niche’) refers to a gradient of vegetation structure ranging from mature forest to grasslands and open fields (see also Table 1).

A species' niche can be described straightforwardly through its position and breadth along well-defined gradients of resources or environmental conditions (Fig. 1, e.g. [6]). A species' niche position usually reflects the average level of a resource that it exploits (or the average climatic condition it copes with). It can therefore be regarded as a coarse-grained measure of resource use, determined by species' evolutionary history and long-term adaptive pressures [7], [8]. Niche breadth (or specialization) corresponds to the range of resource used by a species, i.e. the deviation from its position that it tolerates [9]. Studied together, niche position and niche breadth provide complementary insights into the influence of environmental gradients on species or communities. For instance, the habitat niche position can tell us which vegetation structure is most usually associated with the presence of a given bird species (e.g., mature forest), while its habitat niche breadth indicates the extent to which the species is able to dwell within other structures (e.g. tree plantations or bushy environments). However, niche position and breadth have generally been considered as independent drivers of species' distributions and responses to environmental changes. Notably, habitat specialization, irrespective of habitat position, has been used to predict European birds' sensitivity to land use changes ([10], [11], but see [12]). Similarly, while climatic niches (or envelopes) are defined by both their average climatic position and their climatic breadth, these two variables have seldom been considered together in models of birds' response to climatic changes, except as concurrent predictors (e.g. [13], [14], but see [15]).

Evidence for relationships between climatic and habitat niches remains sparse in the literature. To date, many studies have considered the habitat and climatic components of the ecological niche (hereafter referred to as “habitat niche” and “climatic niche”, Fig. 1) as independent parameters of species' distributional responses to global changes [3]. Hence, species' distributions (and their changes) have usually been explained and predicted through coarse-grained climatic variables, with local habitat being regarded as a secondary fine-grained filter with limited predictive power [16]. However, as the distribution of habitats is partly related to geographical variations in climatic conditions, climate and habitat can also be expected to drive shifts in species' distributions concurrently [17], [18], [19]. Climatic changes influence distributions through processes that occur at very local scales, including local adaptation, step-by-step dispersal, and changes in biotic interactions or in individual fitness [20]. For instance, while climatic conditions may prevent a species from occupying suitable habitats, the same species may also occur in suitable habitats outside the limits of its climatic niche through source-sink dynamics [21]. Although integrating both climatic and habitat variables in species distribution models has a heterogeneous effect on their predictive efficiency [1], [17], [18], it can therefore reasonably be expected that the realized climatic niches of various animal species are to some extent mediated by the distribution of habitats.

We propose hereafter a first investigation of patterns of relationships between habitat and climatic niches in birds, considering both niche positions and breadths. Using data on the spatial distribution of 74 common French breeding bird species, we specifically addressed two questions.

### i – Are habitat and climatic niche positions related?

The relationship between habitat and niche position could be influenced by the fate of species' habitats following past climatic changes. During the last postglacial period, forests recolonized Europe as their lower climatic limits moved northwards [22]. Open vegetation structures (e.g. herbaceous habitats or low forest stages) became spatially scattered across the continent, with no clear latitudinal gradient [22], [23]. Thus, if climatic niches are the primary driver of species distributions at large scales, species with various habitat niche positions should occur in both warm and cold regions. However, recently, more intense wildfire and agricultural pressures in the South led to more heterogeneous landscapes than in the North, although forests were never completely removed at any latitude after the last glaciation [24]. As a result, if species distributions are influenced by the large-scale distribution of their habitats, forest species should exhibit colder climatic positions than open-land species.

### ii – Are habitat and niche breadths related?

We made two competing predictions concerning the correlation between thermal and habitat niche breadths. Brown's niche breadth hypothesis [25] suggests that species should consistently have either broad or narrow breadths on various axes of their ecological niches, because of correlations between gradients of resources or constraints [25]. This first process should result in a positive correlation between habitat and thermal niche breadths. Alternatively, evolutionary cost-benefit trade-offs between efficiently exploiting a particular set of resources and being able to tolerate a wide range of climatic conditions, should drive a negative or nil relationship between habitat and thermal niche breadths (e.g. [26]).

## Methods

### Bird data

**Figure 2:**
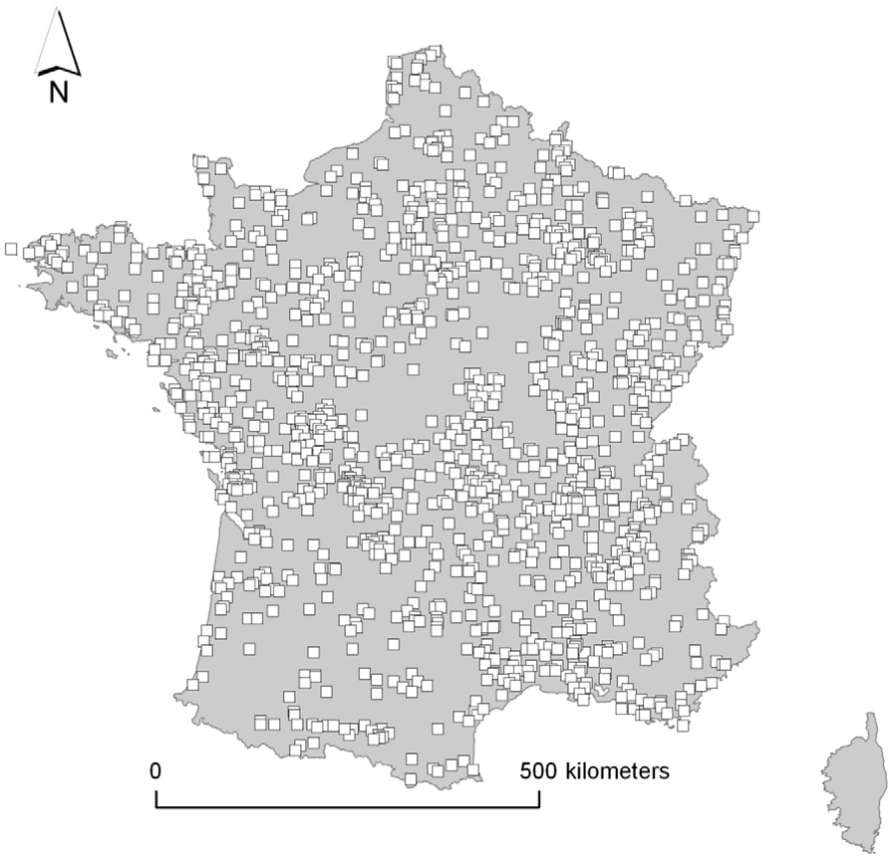
Map of the 1391 FBBS plots surveyed at least once during the period 2002–2008. Each plot consists of a 4 km^2^ square within which the abundances of breeding birds are surveyed through 10 point counts reflecting the local diversity of habitats.

We used data from the French Breeding Bird Survey (FBBS), a long-term monitoring scheme launched in 2001 in which volunteer ornithologists survey common breeding bird species on fixed point counts distributed throughout France (Fig. 2, [27]). We excluded the first survey year, in which data were sparse and spatially clustered, and so we exploited a seven-year survey period (2002 to 2008; 1391 plots surveyed at least one year, 648±96 SD plots per year). In the FBBS, 2×2 km square plots were randomly selected within a 10 km radius around a locality provided by the observer. In each plot, 10 point counts had to be monitored, to represent at best the diversity of occurring habitats. Each year, the observers provided a hierarchical description of the habitat surrounding each point count, from which we derived a simplified habitat classification on an explicit eight-class gradient of habitat structure ranging from forest to farmland (Table 1 and Table S1). We thus facilitated the interpretation of habitat niche positions and breadths by approximating habitat through an ordinal variable reflecting a gradient whose influence on European bird distributions is well known [28]. Points that could not be classified along this gradient due to insufficient habitat description were excluded, resulting in an average of 2997±847 points per survey year. No latitudinal trend appeared in the distribution of habitats at the FBBS scale, but forests were proportionally more abundant in colder areas, essentially owing to altitudinal climatic gradients (Figures S1, S2, S3, S4).

**Table 1:** Description of the habitat gradient.

Habitat class	Description
1	Mature forest stand
2	Sparse or urban forest
3	Young stand, up to 10 m high
4	Young stand, up to 5 m high
5	Young stand, less than 3 m high
6	Agricultural landscape with tree-planted hedges
7	Agricultural landscape with tree lines without hedges/hedges without trees
8	Agricultural landscape without hedges or trees

Eight habitat classes ranging from mature forests to farmlands were derived from a hierarchical classification of habitats performed at each bird point count by observers (Table S1).

Observers surveyed each point twice a year, once before and once after 8 May, with a 4- to 6-week interval between visits. A visit consisted of a 5-minute count during which all species heard or seen in a radius of 100 meters around the point, except for flyovers, had to be identified and counted. Raptors and wetland species were excluded because they were not adequately sampled by this scheme. We eventually analyzed a sample of 74 species, listed in Table S2. For each species and each point, we averaged the counts between the two visits to prevent over-estimation of the true density, e.g. due to the presence of transient birds, or under-estimation due to non-singing birds at the first visit (late migrants) or the second (early singing species).

#### Habitat niche

We quantified habitat niche positions from birds' habitat-level mean densities at the FBBS scale (for each of the eight habitat classes considered). We limited the potential influence of spatial and temporal variations in niche breadth [29] by computing separate positions in each of the biogeographical zones represented within the FBBS area (Alpine, Atlantic, Continental, Mediterranean, following Bossard et al. [30]) and for each year of the survey period. Concretely, for a species *i*, a year *j*, and a zone *z*, the habitat position index *HPI_i,j,z_* was calculated as the average habitat (*k*) weighted by the species' habitat-level densities *d_i,j,z,k_*, according to
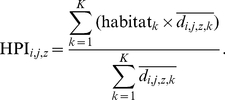
(1)We subsequently averaged *HPI_i,j,z_* across years and zones to obtain a single habitat niche position for each species.

The breadth of the habitat niche reflects the difference between species that are spread across all or many possible habitat structures (generalists) and those restricted to a few habitats (specialists) at large spatial scales. The variance in the densities of a specialist species across a fixed number of habitat types should therefore be lower than that of a generalist [9]. On this assumption, we measured species' habitat breadth through the coefficient of variation of a species' densities across our eight habitat classes (Species Specialization Index, SSI [31]). For a species *i*, in a year *j*, and a zone *z*, the SSI*_i,j,z_* was calculated by
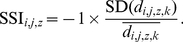
(2)The × −1 coefficient was applied so that the SSI increased with the breadth of the niche, for consistency with the direction of our thermal breadth measure (see below). Like habitat positions, habitat breadths were averaged across years and zones. The habitat niche of each species was therefore described by a single measure of habitat position, and a single measure of habitat breadth.

Although habitat niche metrics may depend to some extent on the landscape context in which they are computed, the SSI has previously been shown to be robust with regard to several sources of heterogeneity, including variations in local habitat composition and less-than-one detectability [32]. FBBS-level computations of HPI and SSI used in our analyses were also well correlated to regional ones computed for each bioclimatic zone (Table S3). This, together with the fact that habitats did not exhibit any strong spatial structure within the FBBS, suggests that the influence of the local context on our analyses was limited.

#### Climatic niche

Like habitats, the climatic niche can be quantified in terms of climate position (the average climate experienced by a species over its current range) and climate breadth (the range of climates that a species tolerates). Consistent with several previous studies, we surrogated the climatic niche by its thermal component, which has been shown to account accurately for the distributions of European birds, and their responses to ongoing climate changes [13], [15]. We thus computed thermal niche positions and breadths for the 74 species of our bird data set. For this purpose, we coupled 0.5×0.5° grids providing the mean March to July monthly temperatures (Wordclim database, http://www/wordclim.org) to species' Western Palaearctic distribution ranges obtained by digitizing maps published by Cramp & Simmons [33]. The thermal position of a species corresponded to the average temperature experienced by the species over its range [34]. The thermal breadth was the difference between the mean temperature of the 5% hottest and 5% coldest grid cells of the species' presence [15]. Both climatic position and breadth were log-transformed to limit the effect of extreme values.

#### Methodological limitations of niche indices

Strong regional variations in species' niches could impair our habitat niche indices, which should ideally be defined over the whole Palaearctic range of a species (like the thermal indices, see above). Unfortunately, standardized habitat-level data on bird abundances are still unavailable at a satisfactory resolution for the Western Palaearctic. The effects of this discrepancy between the scales at which the habitat and thermal niches are computed are explored in Figures S5, S6, S7. Habitat niche indices were robust with regard to a reduction in their scale of computation (from the FBBS to within-France bioclimatic regions), indicating that regional variations in bird habitat niches were rather limited, and thus that our measures of habitat and climatic niches were comparable despite the scale discrepancy. In addition, estimating bird thermal niches at the scale of the FBBS (like the habitat niches) would be irrelevant, as the ranges of most of the species included in our analyses extend far beyond the limits of this particular sampling scheme (with, specifically, no endemics occurring within the FBBS area).

Finally, independently of this issue of scales, niche indices are subject to several sources of measurement error, arising from poor precision and resolution of climatic and atlas data, subjectivity in habitat assignment in the hierarchical coding system of the FBBS, and possible bird sampling biases. As we did not account for such sources of error, the variance of our model parameters may be underestimated [35]. In particular, thermal niche positions and breadths are closely related to species' range size and geographical position. Consequently, thermal breadths are estimated from greater sample sizes, and so more robustly, for widespread species than for geographically restricted ones. This possible discrepancy should, however, have only a limited impact on our results, as all the species included in our data are common and widespread at the Palaearctic scale. Additionally, measuring error in niche indices such as ours comes up against several methodological difficulties. First, the variance of a habitat position is to some extent circular with our measure of habitat breadth. Second, disentangling true measurement errors (from sampling design, observer performance, data resolution, etc.) from ecological sources of niche variability (local adaptation, plasticity), though feasible, is not straightforward in such large-scale data. We see, however, no reason to suppose that the combination of these errors would directionally bias the correlations between niche indices, and so spuriously drive the correlation observed.

### Statistical analyses

Our aim was to assess the extent to which thermal niche positions or breadths were correlated with habitat niche positions or breadths. Such an analysis is liable to be blurred by phylogenetic clustering, because species that share long evolutionary histories are more likely to exhibit similar niches than evolutionary distant ones [36]. We therefore performed our analyses through a phylogenetic generalized least square regression framework (PGLS, [37]), implemented in the libraries ape and nlme in the R software [38], [39]. All niche metrics were standardized to mean = 0 and SD = 1 so that they ranged along comparable scales.

We built two separate PGLS, relating either climatic positions or breadths (responses) to habitat positions or breadths (predictors), respectively. This choice was motivated by our initial prediction that the largest-scale niche (climatic) patterns might be partly driven by the smaller-scale (habitat) ones. We introduced only linear terms into the models, as exploratory analyses did not point to any non-linearities in the relationships between niche parameters. We further included fixed-effect variables to control analyses for species-specific traits likely to influence niche characteristics. At any scale, selection processes impact more strongly on species with long generation times. This should narrow their niche breadths, while species with shorter generation times are expected to have wider niches [40]. Migration strategy also correlates well with species' response to climate and habitat changes [10]. Age of first breeding (age one year or more, as the closest indicator to generation time available for all species in our sample) and migratory status (long- or short-distance migrant) were thus included in models for both niche positions and breadths based on data from Cramp & Simmons [33].

The model structure was therefore formulated as follows:
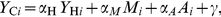
(3)where *Y*_C*i*_ and *Y*_H*i*_ are respectively the climatic and habitat niche parameters (either position or breadth, according to the model), *α* the coefficients of the fixed effects (*M_i_* migratory status, *A_i_* age of first breeding), and *γ* the intercept of the model. We accounted for the phylogenetic relationships between species through a Brownian correlation structure based on a phylogenetic tree with branch length published by Thuiller et al. [41] (Figure S8), to our knowledge the most recent for birds. Note that we obtained very similar results when using other phylogenetic correlation structures (Blomberg, Martins and Grafen correlations, [38]). We built all possible candidate models nested within the model (1), including intercept-only models, and selected models on the basis of Akaike's Information Criterion corrected for small samples (AICc, [42]). We averaged the parameters of all models departing from the best model by less than 2 AICc units, weighting with AICc weights [42]. The uncertainty implied by the model selection procedure is thus incorporated into the estimate of model coefficients.

## Results

**Figure 3:**
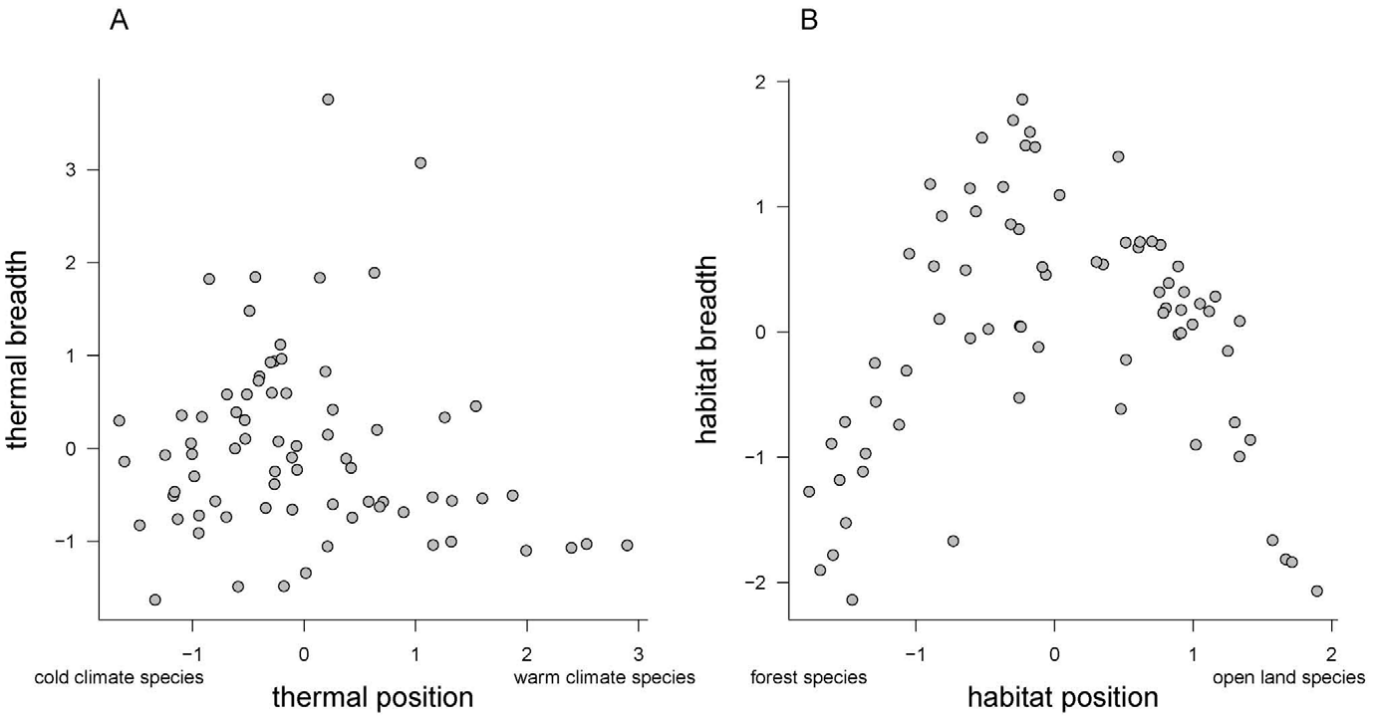
Relationship between position and breadth in each of the niche dimensions considered (A: thermal; B: habitat) for 74 European bird species. Thermal positions and breadths are log-transformed. Variables are scaled to mean = 0, SD = 1 for interpretability.

Our sample of 74 species covered a wide range of thermal position (mean STI = 10.9±1.2°C, range 7.6 to 16.7°C, uncertainties expressed in SD units) and included both thermal specialists and generalists (mean thermal breadth = 14.8±2.6°C, range 10.6 to 24.6°C). Thermal position and breadths were unrelated (Fig. 3A). Habitat positions spread widely along the gradient from forest to farmland species (mean HPI = 4.7±1.6, range 1.8–7.8), with a wide range of habitat breadths (mean SSI = ±1.6±0.5, ranging from −0.7 for the most habitat-generalist species to −2.6 for the most habitat-specialist species). There was a clear convex relationship between habitat position and breadth, with specialists occurring at the extremes of the gradients, while generalists were massed at the centre (Fig. 3B). This relationship is partly due to the way positions were computed, as generalists were forced into middle positions. However, this effect does not impair the rest of our results and its full consideration lies outside our present scope.

**Figure 4:**
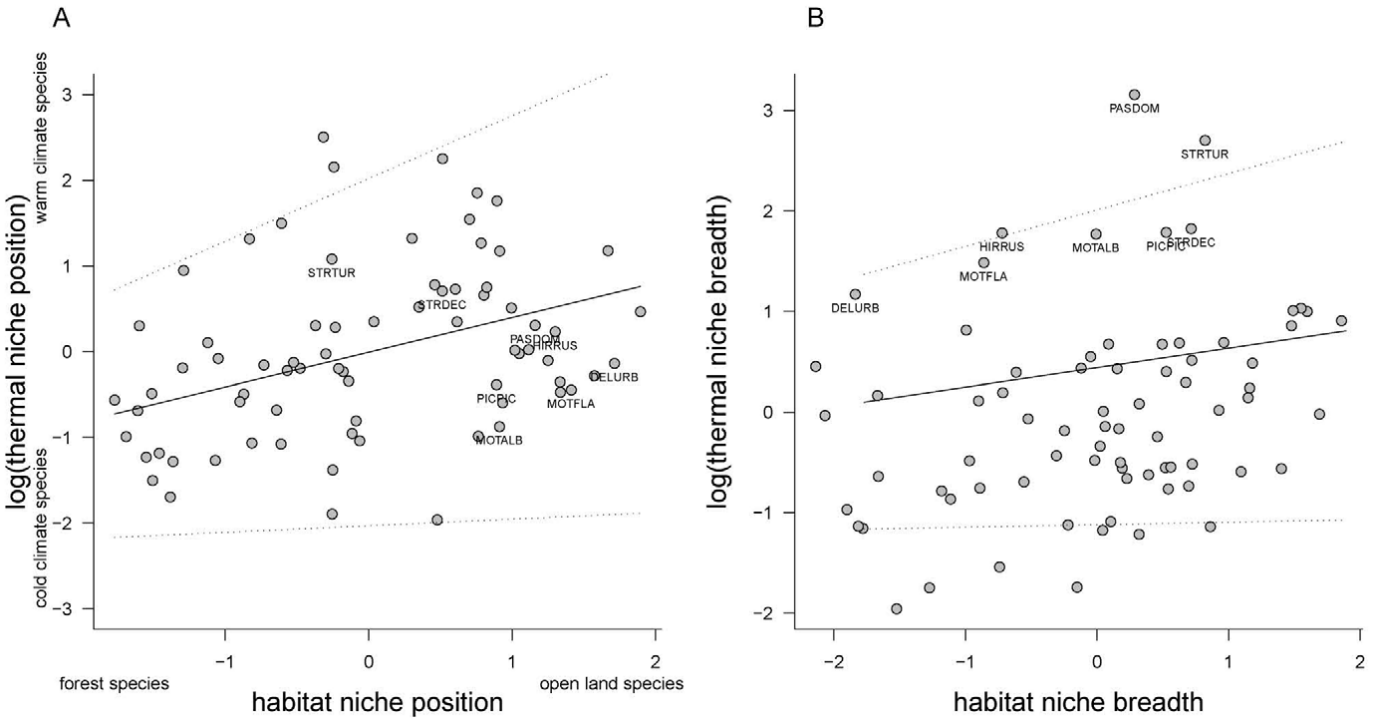
Relationship between thermal and habitat niches for 74 European bird species. (**A**) Relationship between niche positions; (**B**) relationship between niche breadths. The linear relationships (dashed lines) and their confidence intervals (dotted lines) are derived from averaged coefficients resulting from phylogenetic generalized least square regressions, after AICc-based model selection. Thermal positions and breadths are log transformed to approach a normal distribution. Both thermal and habitat positions are scaled to mean = 0, SD = 1. DELURB: *Delichon urbicum*, HIRRUS: *Hirundo rustica*. MOTALB: *Motacilla alba*. MOTFLA: *Motacilla flava*, PASDOM: *Passer domesticus*. PICPIC: *Pica pica*. STRDEC: *Streptopelia decaocto*. STRTUR: *Streptopelia turtur*.

Models explaining climatic niche that included either habitat position or breadths were consistently better than those without habitat niche indices (Table 2), although the relationships appeared noisy (Fig. 4; note that no consensual measure of goodness-of-fit is currently available for such models). Migratory status was retained (Table 2), but its effect was insignificant in both niche position (averaged coefficient for short distance migrants = 0.17±0.28) and niche breadth models (averaged coefficient for short distance migrants = −0.06±0.15). Age of first breeding was removed in both cases (Table 2). Including range size or centroid as additional predictors in maximum models did not remove the effect of habitat niche indices (Table S4); yet, model selections and outputs presented in these results exclude these two predictors due to their structural correlation with thermal niche indices (Figure S9).

**Table 2:** Results of the model selection process.

Response variable: Climatic niche position				
Model (fixed structure)	*k*	AICc	ΔAICc	weight
**γ+HPI**	3	263.00	0.00	0.36
**γ+HPI+migr**	4	263.30	0.34	0.30
γ+HPI+AFB	4	265.20	2.23	0.12
γ+AFB+migr+HPI	5	265.60	2.64	0.10
γ	2	266.50	3.51	0.06
γ+migr	3	267.60	4.63	0.04
γ+AFB	3	268.70	5.67	0.02
γ+migr+AFB	4	269.90	6.85	0.01

Response variable: Climatic niche breadth				
**γ+SSI**	**3**	**225.10**	**0.00**	**0.43**
**γ+SSI+migr**	**4**	**226.60**	**1.57**	**0.19**
γ+SSI+AFB	4	227.30	2.24	0.14
γ	2	227.90	2.89	0.10
γ+AFB+migr+SSI	5	228.90	3.87	0.06
γ+migr	3	230.00	4.98	0.04
γ+AFB	3	230.10	5.06	0.03
γ+migr+AFB	4	232.30	7.21	0.01

Models are phylogenetic generalized least square regressions with either climatic niche position or breadth as the response variable. Fixed predictors included migratory status (migr), age of first breeding (AFB), and either habitat niche position (HPI) or breadth (SSI) according to the model. The intercept is noted *γ*; *k* corresponds to the number of model parameters. The ΔAICc refers to the difference between the AICc of model *i* and that of the model with the lowest AICc value. The column “weight” refers to AICc weights, which were used to compute the averaged coefficients of the fixed effects.

Thermal and habitat positions were positively related (averaged coefficient of habitat position = 0.41±0.16SE, Fig. 4A): the more a species tended to favour forested habitats, the colder was its thermal position. The thermal niche breadth was positively related to habitat niche breadth (averaged coefficient for habitat breadth = 0.19±0.08 SE, Fig. 4B), so that habitat specialists were also thermal specialists.

Eight species had substantially wider thermal niche breadths than the average (mean = 19.85±2.24°C, mean of the remaining 66 species = 13.91±1.61°C, one-sided mean comparison test *t* = −7.27, df = 7.90, *p*<0.0001; Fig.4B). Notably, six of these eight climatic generalists were those most closely linked to human settlements in our species sample (*Streptopelia decaocto*, *Hirundo rustica*, *Delichon urbicum*, *Motacilla alba*, *Pica pica* and *Passer domesticus*) [33].

## Discussion

We found patterns of correlation between the thermal and habitat niches in 74 common European bird species, for both niche positions and breadths. Although high variability impaired these relationships, they were robust with regard to model selection and to the inclusion of phylogeny and life history traits in the models.

Our results first suggest that birds' realized thermal niches are to a certain extent mediated by the climatic distribution of their habitats. Following the last glacial period, temperate plant species recolonized the whole European continent from southern refugia [22], [23]. Forest bird species may therefore have been able to track the northward extension of their habitat because of their thermal plasticity or through local adaptation, until they reached the limits of their physiological tolerance [22] or other (possibly non-climatic) barriers to dispersal [43]. By contrast, open-habitat species exhibit warmer positions despite the presence of open habitats in the North. This suggests that higher proportions of open lands in the South, partly mediated by fire disturbance regimes and agriculture, may have constrained the realized thermal niches of these species more than the climatic influences. Alternatively, and contrasting with our first interpretation, these species' habitat niches may have been first constrained by the distribution of suitable warm climates. Note however that our study does not encompass a few boreal species occurring above the latitudinal limits of tree range, which are related to extremely open habitats.

The extent to which the biogeographic context and the scale of our study could mask more complex relationship between habitat and climatic niches depends on the number and abundance of such extreme species, but does not necessarily influence hypotheses on the underlying processes. Additionally, the high level of noise found in the correlation between habitat and thermal niche positions may have several causes. First, some bird species may not occur throughout the full latitudinal range of their preferential habitat owing to limitations to dispersal and/or the influence of other resources that mediate latitudinal gradients in habitat quality. Second, some species with opposite habitat positions have similarly large thermal breadths. Unlike more climatically bounded species, the northern limit of such species' ranges may have tracked the postglacial northward recolonization of their habitats, without a simultaneous change in their southern limits. This is particularly likely for some forest species such as *Dendrocopos major* (*HPI* = 3.6, thermal breadth = 16.3°C), and farmland specialists with similarly broad climatic requirements such as *Alauda arvensis* (*HPI* = 6.8, thermal breadth = 16.3°C).

We also found that species with narrow thermal breadths were also more often habitat specialists. This pattern sustains the hypothesis that birds' realized thermal niches are at least partly determined by past and current climatic influences on habitats, in conjunction with birds' climatic tolerance itself. Thermal specialists may be restricted to the particular habitat structures prevailing within their thermal ranges because climatic constraints prevent local adaptation or dispersal at their range margins, and so isolate them from new habitat conditions [44], [45], [46]. By contrast, species whose colonization ability is not limited by climatic constraints can spread across wider areas and encounter, on average, a wider diversity of habitat. Such species are therefore more prone to habitat generalism, either because of plastic habitat selection or local adaptation to differing habitats at differing climatic locations within the range [47], [48]. Alternatively, narrow habitat breadths may be a barrier to filling the entire climatic space available to a species [43], in which case a species' distribution would be constrained more by the distribution of its optimum habitat than by climate itself. This constraint may arise from climatic influences on habitat gradients, but also from human land use gradients, correlated with climate without a direct underlying process [18]. Such a human-mediated alteration of birds' distributions is sustained by the broader thermal breadths exhibited by human-related species in our data. However, this non-climatic factor is unlikely to be the only driver of our results. Indeed, although continental scale patterns of land use mediate more heterogeneous habitats in southern Europe than in the North [24], none of the habitat structures considered in our study has ever been completely eliminated from an entire region due to human influence.

The role of climate in shaping the distributions of European birds has previously been demonstrated against neutral models [49], which showed that their climatic niches are not merely an artefact of the regional distribution of climates. However, because climatic gradients are geographically structured in the Western Palaearctic, species' ranges are intrinsically related to their climatic niches. Hence, the transferability of the correlation that we observed with European birds remains to be tested. Beyond this point, correlations between habitat and climatic niches may vary within species' ranges, in relation with climatic gradients [48] or other factors that contribute to shape the breadth of realized niches, including changes in interspecific interactions. Theoretical approaches predict that species should become increasingly tied to their optimum resources as approaching the borders of their distributions, due to resource instability and/or lower abundance [20]. Hence, the correlation between habitat and climatic niches is expected to decrease near a species' range limits, as the influence of local resource availability becomes stronger compared with larger-scale factors. Exploring such spatial variation in the relationships between species ‘climatic and habitat niches would be a significant step forwards to our understanding of range setting. In this respect, a null-modeling framework combined with fine-grained data on habitat requirements could help disentangling pure sampling effect from evolutionary and ecological processes.

### Conclusion

Distribution models often show that climatic variables predict species distributions better than habitat or land use variables, but the underlying causes remain unclear [50] and are scale-dependent [16]. Our results argue for considering mutual influences of habitat and climatic niche on each other, questioning the extent to which species' responses to climatic gradients should be attributed to direct processes or to changes in habitats correlated with changes in climate.

Because large scale multispecies analyses like ours rely on correlative patterns, we do not claim that our results formally demonstrate any underlying process (either that habitats are primary drivers of the realized climatic niche or that climatic suitability has constrained habitat niches). However, range dynamics are mediated by local processes [20]. We therefore believe it reasonable to state that the correlation between the two niche dimensions indicates that habitat concurs with climate in shaping species' distributional responses to climatic gradients, within the limits of their physiological thermal tolerance. This does not necessarily imply that the habitat and climatic niches are evolutionarily related, in which case the pattern of correlation that we observe would be maintained in areas where climate is not a primary driver of the distribution of habitat structures. Even so, our results suggest that instead of being merely a fine-scale filter, the habitat niche could contribute to shape the climatic distributions of bird species. Simultaneously, despite usually regarded as a coarse-grained filter, climate could be an influential component of species' small-scale responses to habitat gradients. Because local adaptation, biotic interactions and dispersal are essential mechanisms of range limit settings [51], and so depend directly on local habitat gradients, large-scale climatic distributions could be influenced by local processes directly impacting on population dynamics and selection processes [52]. It follows that ongoing climate changes would only affect distributions if new climatically suitable areas also underwent habitat changes matching species' habitat niche position. Such process could contribute to discrepancies between occurring shifts in species' distributions and those predicted by their realized climatic niches [34]. In this respect, a fuller understanding of the consequences of within-niche relationships will undoubtedly allow a major advance towards efficient predictive and mechanistic range modelling.

## Supporting Information

Figure S1 Number of FBBS points per degree of latitude for each habitat class. The habitat classes are described in Table S1b. Habitats are ordered from the most forested one (1) to the most open one (8).
(DOCX)

Figure S2 Proportion of points of each habitat class within FBBS plots, per degree of latitude. The proportions are computed as the percentage of points of habitat class X, relative to the total number of points within a 4 km^2^ plot (10 points in each plot). The habitat classes are described in Table S1b. Habitats are ordered from the most forested one (1) to the most open one (8).
(DOCX)

Figure S3 Proportion of points of each habitat in FBBS squares, with respect to minimum temperatures in the square. Temperatures are averaged from monthly minimum temperatures over 1971–2000 (data from the French center for Meteorology, Météo France). The habitat classes are described in Table S1b. Habitats are ordered from the most forested one (1) to the most open one (8).
(DOCX)

Figure S4 Proportion of points of each habitat in FBBS squares, with respect to maximum temperatures in the square. Temperatures are averaged from monthly maximum temperatures over 1971–2000 (data from the French center for Meteorology, Météo France). The habitat classes are described in Table S1b. Habitats are ordered from the most forested one (1) to the most open one (8).
(DOCX)

Figure S5 Correlations between habitat breadths computed at the scale of the FBBS area (x axis) and habitat breadths computed for each biogeographic zone of the FBBS.
(DOCX)

Figure S6 Correlations between habitat positions computed at the scale of the FBBS area (x axis) and habitat positions computed for each biogeographic zone of the FBBS.
(DOCX)

Figure S7 Correlations between thermal positions or thermal ranges between three spatial scales: Western Palaearctic, Europe, FBBS. Pearson's R-squared are provided above each plot. In this analysis, we correlate climatic niche positions and breadths computed at three different spatial scales, for the 74 species accounted for in the main analyses. The “Palaearctic scale” is the scale used for the main analyses. The data at the “Europe” scale are extracted from [34]. Finally, French Breeding Bird Survey (FBBS)- scale climatic positions and breadths were computed using the same data as used for the habitat niche computations in the main text.
(DOCX)

Figure S8 Phylogenetic tree of the 74 species listed in Table S2.
(DOCX)

Figure S9 Correlation between (A) thermal position and latitude of range centroid; (B) thermal breadth and range size. N = 74 species for both relationships, the names of outlying species are indicated in (A).
(DOCX)

Table S1 Habitat classes used for the computation of niche parameters. Table S1a. Original habitat coding system used by observers. Table S1b. Correspondence between the coding system retained in our habitat structure gradient and the original coding system (Table S1a).
(DOCX)

Table S2 List of the 74 bird species, with life history traits, number of occurrences in the FBBS, and range size. Migratory status is either “short distance migrant” (sd) or “long distance migrant” (ld). Sedentary species are counfounded with short distance migrants. Age of first breeding is a two-level variable (1 = first breeding in first year, 2 = first breeding in second year or later). Species are ordered according to the phylogeny used in the analyses (see Figure S5).
(DOCX)

Table S3 Number of points per year/biogeographic zone. Note that each FBBS plot consists in a 4 km^2^ plot including 10 points. Some points were removed when the habitat description was not sufficient to be assigned to one of the eight classes of our habitat gradient (described in Table S1).
(DOCX)

Table S4 Effect of including range size and position as a predictor in models relating thermal and habitat niches. The table shows the statistical significance of predictors in the two model tested (through F tests), together with coefficient estimates for continuous variables.Model structures are the same as the maximum models presented in the Methods section of the main text. Predictors (all scaled to mean = 0, SD = 1) were added as follows: - For the model relating thermal and habitat position, we included the latitude of species' range centroid. - For the model relating thermal and habitat breadths, we included the total area of species' ranges (from the same data as those used to compute thermal indices, see Methods in the main text). We did not perform model selection and averaging with these models due to the structural relationship between range and thermal niche described in the main text, which could artificially obscure the effects of other ecologically important predictors (whether related or not to habitat niche).
(DOCX)
