# Supplementary material for: Relating Habitat and Climatic Niches in Birds
Source: PLoS One. 2012 Mar 12;7(3):e32819. doi: 10.1371/journal.pone.0032819 (PMC3299694; doi:10.1371/journal.pone.0032819)
Supplement: Figure S1 — Number of FBBS points per degree of latitude for each habitat class. The habitat classes are described in Table S1b. Habitats are ordered from the most forested one (1) to the most open one (8). (DOCX) [file pone.0032819.s001.docx]

**Figure S1.** **Number of FBBS points per degree of latitude for each habitat class.** The habitat classes are described in Table S1b. Habitats are ordered from the most forested one (1) to the most open one (8).
