# Supplementary material for: Relating Habitat and Climatic Niches in Birds
Source: PLoS One. 2012 Mar 12;7(3):e32819. doi: 10.1371/journal.pone.0032819 (PMC3299694; doi:10.1371/journal.pone.0032819)
Supplement: Figure S2 — Proportion of points of each habitat class within FBBS plots, per degree of latitude. The proportions are computed as the percentage of points of habitat class X, relative to the total number of points within a 4 km2 plot (10 points in each plot). The habitat classes are described in Table S1b. Habitats are ordered from the most forested one (1) to the most open one (8). (DOCX) [file pone.0032819.s002.docx]

**Figure S2.** **Proportion of points of each habitat class within FBBS plots, per degree of latitude.** The proportions are computed as the percentage of points of habitat class X, relative to the total number of points within a 4 km² plot (10 points in each plot). The habitat classes are described in Table S1b. Habitats are ordered from the most forested one (1) to the most open one (8).
