# Supplementary material for: Relating Habitat and Climatic Niches in Birds
Source: PLoS One. 2012 Mar 12;7(3):e32819. doi: 10.1371/journal.pone.0032819 (PMC3299694; doi:10.1371/journal.pone.0032819)
Supplement: Figure S4 — Proportion of points of each habitat in FBBS squares, with respect to maximum temperatures in the square. Temperatures are averaged from monthly maximum temperatures over 1971–2000 (data from the French center for Meteorology, Météo France). The habitat classes are described in Table S1b. Habitats are ordered from the most forested one (1) to the most open one (8). (DOCX) [file pone.0032819.s004.docx]

**Figure S4. Proportion of points of each habitat in FBBS squares, with respect to maximum temperatures in the square.** Temperatures are averaged from monthly maximum temperatures over 1971-2000 (data from the French center for Meteorology, Météo France). The habitat classes are described in Table S1b. Habitats are ordered from the most forested one (1) to the most open one (8).
