# Supplementary material for: Relating Habitat and Climatic Niches in Birds
Source: PLoS One. 2012 Mar 12;7(3):e32819. doi: 10.1371/journal.pone.0032819 (PMC3299694; doi:10.1371/journal.pone.0032819)
Supplement: Figure S5 — Correlations between habitat breadths computed at the scale of the FBBS area (x axis) and habitat breadths computed for each biogeographic zone of the FBBS. (DOCX) [file pone.0032819.s005.docx]

**Figure S5. Correlations between habitat breadths computed at the scale of the FBBS area (x axis) and habitat breadths computed for each biogeographic zone of the FBBS.**
