# Supplementary material for: Relating Habitat and Climatic Niches in Birds
Source: PLoS One. 2012 Mar 12;7(3):e32819. doi: 10.1371/journal.pone.0032819 (PMC3299694; doi:10.1371/journal.pone.0032819)
Supplement: Figure S7 — Correlations between thermal positions or thermal ranges between three spatial scales: Western Palaearctic, Europe, FBBS. Pearson's R-squared are provided above each plot. In this analysis, we correlate climatic niche positions and breadths computed at three different spatial scales, for the 74 species accounted for in the main analyses. The “Palaearctic scale” is the scale used for the main analyses. The data at the “Europe” scale are extracted from [34]. Finally, French Breeding Bird Survey (FBBS)- scale climatic positions and breadths were computed using the same data as used for the habitat niche computations in the main text. (DOCX) [file pone.0032819.s007.docx]

**Figure S7. Correlations between thermal positions or thermal ranges between three spatial scales:** Western Palaearctic, Europe, FBBS. Pearson’s R-squared are provided above each plot. In this analysis, we correlate climatic niche positions and breadths computed at three different spatial scales, for the 74 species accounted for in the main analyses. The “Palaearctic scale” is the scale used for the main analyses. The data at the “Europe” scale are extracted from [34]. Finally, French Breeding Bird Survey (FBBS)- scale climatic positions and breadths were computed using the same data as used for the habitat niche computations in the main text.
