# Supplementary material for: Relating Habitat and Climatic Niches in Birds
Source: PLoS One. 2012 Mar 12;7(3):e32819. doi: 10.1371/journal.pone.0032819 (PMC3299694; doi:10.1371/journal.pone.0032819)
Supplement: Figure S8 — Phylogenetic tree of the 74 species listed in Table S2. (DOCX) [file pone.0032819.s008.docx]

**Figure S8. Phylogenetic tree of the 74 species listed in Table S2.**
