# Supplementary material for: Relating Habitat and Climatic Niches in Birds
Source: PLoS One. 2012 Mar 12;7(3):e32819. doi: 10.1371/journal.pone.0032819 (PMC3299694; doi:10.1371/journal.pone.0032819)
Supplement: Figure S9 — Correlation between (A) thermal position and latitude of range centroid; (B) thermal breadth and range size. N = 74 species for both relationships, the names of outlying species are indicated in (A). (DOCX) [file pone.0032819.s009.docx]

**Figure S9. Correlation between (A) thermal position and latitude of range centroid; (B) thermal breadth and range size.** N=74 species for both relationships, the names of outlying species are indicated in (A).
