# Supplementary material for: Relating Habitat and Climatic Niches in Birds
Source: PLoS One. 2012 Mar 12;7(3):e32819. doi: 10.1371/journal.pone.0032819 (PMC3299694; doi:10.1371/journal.pone.0032819)
Supplement: Table S1 — Habitat classes used for the computation of niche parameters. Table S1a. Original habitat coding system used by observers. Table S1b. Correspondence between the coding system retained in our habitat structure gradient and the original coding system (Table S1a). (DOCX) [file pone.0032819.s010.docx]

Table S1: Habitat classes used for the computation of niche parameters

Table S1a. Original habitat coding system used by observers

Class

Sub-category 1 sub-category 2 sub-category 3

**A**. Forest (trees > 5m high)

1 deciduous 1 semi-natural or mixed ages 1 Oak

2 conifer 2 even-aged plantation 2 Beech

3 mixed (> 10% each) 3 uneven-aged plantation 3 Maple

4 mature plantation (> 10m high) 4 Chestnut tree

5 young plantation (5-10m) 5 other deciduous

6 park (sparse trees with meadows) 6 Fir

7 dense undergrowth 7 Spruce

8 moderate undergrowth 8 Pine

9 sparse undergrowth 9 Larch

10 presence of deadwood 10 other conifer

11 absence of deadwood 11 other species

**B**. Bushes (or young forest < 5m high)

1 regeneration forest 1 deciduous 1 mostly high (3-5 metres)

2 chalky bushes 2 conifer 2 mostly small (1-3 metres)

3 moorland 3 mixed (10% each) 3 dense undergrowth

4 young coppice 4 marsh deciduous bushes 4 moderate undergrowth

5 new plantation 5 marsh coniferous bushes 5 sparse undergrowth

6 clear cut 6 marsh mixed bushes 6 fern

7 others 7 evergreen leaves 7 pasture

8 garrigue

9 scrubland

# **C**. grasslands, marshes and moorlands

1 dry calcareous grassland 1 edge with trees 1 non pastured

2 herbaceous moorland 2 edge without trees 2 Pastured

3 heather moorland 3 tree line without edge 3 hay

4 natural wet grassland 4 other field limit (wall, ditch) 4 many ferns

5 other dry grasslands 5 isolated trees (1 – 10)

6 flooded grassland /pastured marsh 6 no edge nor limit

7 reed bed 7 Mountain

8 other open marshes 8 dyke

9 exploited salt marshes

10 peat bog

11 natural salt marshes

# **D**. cultivated areas

1 cultivated meadow 1 edge with trees 1 non pastured

2 non cultivated meadow 2 edge without trees 2 pastured

3 mixed meadows - crops 3 tree line without edge 3 cereals

4 large scale crops 4 other field limit (wall/ditch)4 maize

5 grove / grapevine / truckers 5 isolated trees (1 – 10) 5 sunflowers

6 other crops 6 farmyard 6 rape

7 no edge nor limit 7 root crops

8 naked soil

9 other crops

10 rice fields

Table S1b. Correspondence between the coding system retained in our habitat structure gradient and the original coding system (Table S1a)

| **category** | **broad class** | **sub - category 1** | **sub - category 2** | **sub - category 3** |
| --- | --- | --- | --- | --- |
| 1 | A | 1 - 3 | 1 - 4 | 1 - 11 |
| 2 | A | 1 - 3 | 6 | 1 - 11 |
| 3 | A | 1 - 3 | 5 | 1 - 11 |
| 4 | B | 1 - 7 | 1 - 9 | 1 |
| 5 | B | 1 - 7 | 1 - 9 | 2 |
| 6 | C | 1 - 11 | 1 | 1 - 4 |
|  | D | 1 - 6 | 1 | 1 - 10 |
| 7 | C | 1 - 11 | 2 - 3 | 1 - 4 |
|  | D | 1 - 6 | 2 - 3 | 1 - 10 |
| 8 | C | 1 - 11 | 4 - 8 | 1 - 10 |
|  | D | 1 - 6 | 4 -8 | 1 - 10 |
