# Supplementary material for: Relating Habitat and Climatic Niches in Birds
Source: PLoS One. 2012 Mar 12;7(3):e32819. doi: 10.1371/journal.pone.0032819 (PMC3299694; doi:10.1371/journal.pone.0032819)
Supplement: Table S2 — List of the 74 bird species, with life history traits, number of occurrences in the FBBS, and range size. Migratory status is either “short distance migrant” (sd) or “long distance migrant” (ld). Sedentary species are counfounded with short distance migrants. Age of first breeding is a two-level variable (1 = first breeding in first year, 2 = first breeding in second year or later). Species are ordered according to the phylogeny used in the analyses (see Figure S5). (DOCX) [file pone.0032819.s011.docx]

**Table S2. List of the species included in our analyses and associated life history traits.**

Migratory status is either “short distance migrant” (sd) or “long distance migrant” (ld). Sedentary species are counfounded with short distance migrants. Age of first breeding is a two-level variable (1 = first breeding in first year, 2 = first breeding in second year or later). Species are ordered according to the phylogeny used in the analyses (see Figure S5).

| **species** | **mean number of FBBS squares (2002-2008)** | **migratory status** | **age of first breeding** | **range size (×10^6km²)** |
| --- | --- | --- | --- | --- |
| Dendrocopos major | 256.7 | sd | 1 | 37.47 |
| Dryocopus martius | 67.6 | sd | 1 | 29.48 |
| Picus viridis | 263.6 | sd | 1 | 25.83 |
| Upupa epops | 105.4 | ld | 1 | 35.30 |
| Oriolus oriolus | 186.4 | ld | 2 | 32.96 |
| Lanius collurio | 81.6 | ld | 1 | 34.75 |
| Pica pica | 203.4 | sd | 2 | 51.27 |
| Nucifraga caryocatactes | 3.3 | sd | 2 | 11.09 |
| Corvus monedula | 33.0 | sd | 2 | 39.29 |
| Corvus corone | 468.7 | sd | 2 | 6.45 |
| Corvus frugilegus | 53.6 | sd | 2 | 22.99 |
| Garrulus glandarius | 277.1 | sd | 2 | 41.80 |
| Poecile montanus | 18.4 | sd | 1 | 30.44 |
| Poecile palustris | 80.0 | sd | 1 | 19.34 |
| Periparus ater | 64.4 | sd | 1 | 31.50 |
| Cyanistes caeruleus | 351.6 | sd | 1 | 37.90 |
| Lophophanes cristatus | 64.7 | sd | 1 | 24.77 |
| Parus major | 455.6 | sd | 1 | 48.91 |
| Hippolais polyglotta | 165.6 | ld | 1 | 6.99 |
| Aegithalos caudatus | 105.0 | sd | 1 | 33.35 |
| Phylloscopus bonelli | 51.3 | ld | 1 | 5.31 |
| Phylloscopus sibilatrix | 58.6 | ld | 1 | 26.57 |
| Phylloscopus collybita | 440.6 | ld | 1 | 34.10 |
| Phylloscopus trochilus | 47.9 | ld | 1 | 35.51 |
| Sylvia atricapilla | 506.6 | sd | 1 | 32.22 |
| Sylvia borin | 129.1 | ld | 1 | 32.06 |
| Sylvia cantillans | 12.0 | ld | 1 | 6.65 |
| Sylvia melanocephala | 23.0 | sd | 1 | 9.58 |
| Sylvia communis | 226.7 | ld | 1 | 39.07 |
| Sylvia hortensis | 5.0 | ld | 1 | 3.88 |
| Hirundo rustica | 68.1 | ld | 1 | 49.51 |
| Delichon urbica | 8.0 | ld | 1 | 45.80 |
| Alauda arvensis | 290.3 | sd | 1 | 41.72 |
| Lullula arborea | 114.9 | sd | 1 | 28.15 |
| Passer montanus | 29.1 | sd | 1 | 41.41 |
| Passer domesticus | 236.6 | sd | 1 | 63.09 |
| Fringilla coelebs | 519.6 | sd | 1 | 49.77 |
| Coccothraustes coccothraustes | 41.1 | sd | 1 | 25.23 |
| Carduelis chloris | 230.3 | sd | 1 | 40.51 |
| Loxia curvirostra | 7.3 | sd | 1 | 25.01 |
| Serinus citrinella | 3.5 | sd | 1 | 1.11 |
| Serinus serinus | 85.3 | sd | 1 | 21.07 |
| Carduelis carduelis | 155.0 | sd | 1 | 42.70 |
| Carduelis cannabina | 127.7 | sd | 1 | 38.64 |
| Pyrrhula pyrrhula | 50.3 | sd | 1 | 28.25 |
| Emberiza cirlus | 133.0 | sd | 1 | 9.94 |
| Emberiza citrinella | 245.3 | sd | 1 | 35.95 |
| Emberiza hortulana | 7.3 | ld | 1 | 24.37 |
| Anthus campestris | 9.4 | sd | 1 | 18.15 |
| Anthus trivialis | 158.6 | ld | 1 | 31.30 |
| Anthus spinoletta | 2.0 | sd | 1 | 4.49 |
| Motacilla flava | 90.6 | ld | 1 | 38.61 |
| Motacilla alba | 123.7 | sd | 1 | 49.04 |
| Prunella modularis | 198.4 | sd | 1 | 29.45 |
| Turdus viscivorus | 174.0 | sd | 1 | 35.93 |
| Turdus merula | 528.1 | sd | 1 | 40.86 |
| Turdus torquatus | 5.7 | sd | 1 | 5.81 |
| Turdus philomelos | 371.3 | sd | 1 | 39.67 |
| Sturnus vulgaris | 274.7 | sd | 1 | 41.53 |
| Phoenicurus ochruros | 131.1 | sd | 1 | 19.64 |
| Phoenicurus phoenicurus | 58.3 | ld | 1 | 38.40 |
| Saxicola torquata | 179.1 | sd | 1 | 20.06 |
| Luscinia megarhynchos | 288.3 | ld | 1 | 18.79 |
| Erithacus rubecula | 406.4 | sd | 1 | 35.61 |
| Sitta europaea | 206.6 | sd | 1 | 31.76 |
| Troglodytes troglodytes | 417.4 | sd | 1 | 32.89 |
| Certhia brachydactyla | 225.4 | sd | 1 | 12.91 |
| Certhia familiaris | 21.0 | sd | 1 | 27.00 |
| Regulus ignicapillus | 91.1 | sd | 1 | 11.60 |
| Regulus regulus | 73.6 | sd | 1 | 25.37 |
| Cuculus canorus | 365.6 | ld | 2 | 48.65 |
| Columba palumbus | 461.0 | sd | 1 | 39.68 |
| Streptopelia decaocto | 243.3 | sd | 1 | 32.30 |
| Streptopelia turtur | 272.9 | ld | 1 | 51.84 |
