# Supplementary material for: Relating Habitat and Climatic Niches in Birds
Source: PLoS One. 2012 Mar 12;7(3):e32819. doi: 10.1371/journal.pone.0032819 (PMC3299694; doi:10.1371/journal.pone.0032819)
Supplement: Table S3 — Number of points per year/biogeographic zone. Note that each FBBS plot consists in a 4 km2 plot including 10 points. Some points were removed when the habitat description was not sufficient to be assigned to one of the eight classes of our habitat gradient (described in Table S1). (DOCX) [file pone.0032819.s012.docx]

**Table S3. Number of points per year / biogeographic zone.** Note that each FBBS plot consists in a 4km² plot including 10 points. Some points were removed when the habitat description was not sufficient to be assigned to one of the eight classes of our habitat gradient (described in Table S1).

| **bioclimatic zone** | **mean point number (2002-2008) ±SD** |
| --- | --- |
| FBBS | 6289.1±1076 |
| Alpine | 278.7±107.4 |
| Atlantic | 2748.7±672.8 |
| Continental | 2589.1±471.4 |
| Mediterranean | 672.4±95.5 |
