# Supplementary material for: Relating Habitat and Climatic Niches in Birds
Source: PLoS One. 2012 Mar 12;7(3):e32819. doi: 10.1371/journal.pone.0032819 (PMC3299694; doi:10.1371/journal.pone.0032819)
Supplement: Table S4 — Effect of including range size and position as a predictor in models relating thermal and habitat niches. The table shows the statistical significance of predictors in the two model tested (through F tests), together with coefficient estimates for continuous variables.Model structures are the same as the maximum models presented in the Methods section of the main text. Predictors (all scaled to mean = 0, SD = 1) were added as follows: - For the model relating thermal and habitat position, we included the latitude of species' range centroid. - For the model relating thermal and habitat breadths, we included the total area of species' ranges (from the same data as those used to compute thermal indices, see Methods in the main text). We did not perform model selection and averaging with these models due to the structural relationship between range and thermal niche described in the main text, which could artificially obscure the effects of other ecologically important predictors (whether related or not to habitat niche). (DOCX) [file pone.0032819.s013.docx]

**Table S4. Effect of including range size and position as a predictor in models relating thermal and habitat niches.**

| **RESPONSE: THERMAL POSITION** | | | | |
| --- | --- | --- | --- | --- |
| **predictor** | **estimate±SE** | **df;N** | **F-value** | **p-value** |
| Intercept | 0.15±0.35 | 1;74 | 0.06 | 0.81 |
| migr | - | 1;74 | 9.27 | 0.00 |
| AFB | - | 1;74 | 0.13 | 0.72 |
| HPI | 0.06±0.06 | 1;74 | 54.41 | <.0001 |
| latitude range centroid | -0.95±0.04 | 1;74 | 527.10 | <.0001 |
|  |  |  |  |  |
| **RESPONSE: THERMAL BREADTH** | | | | |
| Intercept | 0.37±0.76 | 1;74 | 0.47 | 0.50 |
| migr | - | 1;74 | 0.10 | 0.76 |
| AFB | - | 1;74 | 0.00 | 0.95 |
| SSI | 0.15±0.08 | 1;74 | 6.24 | 0.01 |
| range size | 0.33±0.08 | 1;74 | 14.43 | >0.001 |

**The table shows the statistical significance of predictors in the two model tested (through F tests), together with coefficient estimates for continuous variables.** Model structures are the same as the maximum models presented in the Methods section of the main text. Predictors (all scaled to mean = 0, SD=1) were added as follows:

- For the model relating thermal and habitat position, we included the latitude of species’ range centroid
- For the model relating thermal and habitat breadths, we included the total area of species’ ranges (from the same data as those used to compute thermal indices, see Methods in the main text).

We did not perform model selection and averaging with these models due to the structural relationship between range and thermal niche described in the main text, which could artificially obscure the effects of other ecologically important predictors (whether related or not to habitat niche).
